# Structure–Property Relationships in Shape Memory Metallic Glass Composites

**DOI:** 10.3390/ma12091419

**Published:** 2019-05-01

**Authors:** Daniel Şopu, Xudong Yuan, Franco Moitzi, Mihai Stoica, Jürgen Eckert

**Affiliations:** 1 Fachgebiet Materialmodellierung, Institut für Materialwissenschaft, Technische Universität Darmstadt, Otto-Berndt-Straße 3, D-64287 Darmstadt, Germany; 2 Erich Schmid Institute of Materials Science, Austrian Academy of Sciences, Jahnstraße 12, A-8700 Leoben, Austria; Xudong.Yuan@oeaw.ac.at (X.Y.); franco.moitzi@stud.unileoben.ac.at (F.M.); juergen.eckert@unileoben.ac.at (J.E.); 3 Laboratory of Metal Physics and Technology, Department of Materials, ETH Zurich, 8093 Zurich, Switzerland; mihai.stoica@mat.ethz.ch; 4 Department of Materials Science, Mountanuniversität Leoben, Jahnstraße 12, A-8700 Leoben, Austria

**Keywords:** metallic glass composites, shape memory alloys, molecular dynamics, plasticity

## Abstract

Metallic glass composites with shape memory crystals show enhanced plasticity and work-hardening capability. We investigate the influence of various critical structural aspects such as, the density of crystalline precipitates, their distribution and size, and the structural features and intrinsic properties of the phase on the deformation behavior of metallic amorphous Cu${}_{64}$Zr${}_{36}$ composites with B2 CuZr inclusions using molecular dynamics simulations. We find that a low density of small B2 inclusions with spacing smaller than the critical shear band length controls the formation and distribution of plastic zones in the composite and hinders the formation of critical shear bands. When the free path for shearing allows the formation of mature shear bands a high volume fraction of large B2 precipitates is necessary to stabilize the shear flow and avoid runaway instability. Additionally, we also investigate the deformation mechanism of composites with pure copper crystals for comparison, in order to understand the superior mechanical properties of metallic glass composites with shape memory crystals in more detail. The complex and competing mechanisms of deformation occurring in shape memory metallic glass composites allow this class of materials to sustain large tensile deformation, even though only a low-volume fraction of crystalline inclusions is present.

## 1. Introduction

Improving the plastic deformability of metallic glasses (MGs) is the key to the extensive use of these attractive materials in structural and functional applications. Hence, understanding and controlling the deformation mechanisms of MGs is a hot research topic and remains a major ongoing issue. The most common strategy to improve the ductility of MGs is the fabrication of MG composites with a crystalline phase [1,2,3,4,5,6,7]. When loaded, the soft crystalline phase yields first and perturbs the strain/stress field in the adjacent glass matrix around the inclusions, thus, promoting the formation of a highly organized pattern of multiple shear bands. Moreover, the crystalline phase arrests shear band propagation, stabilizes the shear flow and avoids runaway instability [8,9]. However, to prevent the formation of critical shear bands and for obtaining tensile ductility the crystalline inclusions must be sufficiently large and, thus, must occupy an overall volume fraction larger than 40 vol.% [10,11,12,13]. Moreover, a high volume fraction of soft precipitates improves the ductility of the MG composites but may significantly lower their strength [14,15]. Besides, MG composites reinforced with a B2-type shape memory phase show extraordinary plasticity together with work hardening capability [7,16,17,18,19,20,21,22]. Here, the deformation-induced martensitic phase transformation of B2 to B19${}^{\prime }$ provides a high density of interfaces of phases and twins [8]. This hardens the crystalline phase and hinders shear bands to penetrate through the precipitates [18,23,24]. Allover, the martensitic transformation in shape memory crystals counterbalances shear-induced softening and localization in the glassy matrix [7].

Molecular dynamics (MD) simulations have provided an atomistic description of the deformation mechanism in MG composites with a B2 shape memory phase [25,26]. An autocatalytic chain-type deformation mechanism of MG composites was found for such composites: the martensitic transformation leads to shear band formation and likewise the stress at the shear band tip induces martensitic transformation in the shape memory crystal, promoting a heterogeneous distribution of strain into the glassy matrix [25,26]. Additionally, the martensitic transformation changes the shear band morphology and leads to a shear-broadening effect that lowers the elastic shear stress and reduces the probability for catastrophic fracture of the composite [26]. The complex and competing mechanisms of deformation occurring in the glassy matrix and B2 inclusions allow amorphous-crystalline nanolaminates with low-volume fraction of B2 phase (below 45 vol.%) to exhibit large tensile ductility and nearly ideal plastic flow behavior. Hence, improved ductility is also expected for MG composites with a low-volume fraction of B2 inclusions.

In this paper, we present MD computer simulations of the deformation processes in MG composites with B2 CuZr crystalline precipitates. In our previous work, the deformation behavior and the underlying deformation mechanism of only one type of MG composite have been investigated [25]. Here, we provide a systematic investigation of the mechanical properties of shape memory MG composites and quantify the influence of different structural aspects on the deformation mechanisms. First, the impact of the volume fraction of the B2 phase on strain localization is investigated. Then, the critical size of the B2 inclusions for which the MG composite shows a brittle-to-ductile transition is identified. Finally, the superior mechanical properties of MG composites with shape memory crystals are highlighted by comparing their deformation mechanisms to those observed for composites with nanocrystalline inclusions which deform via dislocation activity [27,28].

## 2. Simulation Details

For studying the mechanical properties of MG composites with shape memory crystals, classical MD simulations were performed using the program package LAMMPS [29]. A glassy system of 8000 atoms was prepared by quenching from the melt with a cooling rate of 10${}^{10}$ K/s at zero pressure. The interatomic interactions were described by the modified Finnis-Sinclair type potential for CuZr binary alloys proposed by Mendelev et al. [30]. The potential reproduces the stress-induced martensitic transformation in the CuZr B2 crystalline phase that transforms to an intermediate R-phase and finally to the body-centered-tetragonal (BCT) phase under tensile loading [31,32]. Although the Mendelev potential does not properly describe the experimental phase transformation in B2 CuZr crystallites (from a B2 to a B19’ structure [17]), our simulations are still able to provide a reasonable mechanistic and atomistic view of the deformation processes in MG composites reinforced with shape memory precipitates on a qualitative level. Since the chosen EAM-potential by Mendelev massively overestimates the unstable stacking-fault energy and the critical stress for homogeneous dislocation nucleation in copper crystals [27], we used the potential developed by Ward et al. [33] to investigate the deformations mechanisms in MG composites with copper precipitates.

For simulating the deformation mechanism, slab-shaped Cu${}_{64}$Zr${}_{36}$ glass samples with dimensions of $L_{x}$× $L_{y}$× $L_{z}$ = 33.5 × 7.5 × 85.0 nm${}^{3}$ and about 1.3 million atoms were constructed by replicating the initial quenched glassy system. MG composites were constructed by inserting different numbers of B2 CuZr rectangular inclusions with different sizes in the monolithic glass. Afterwards, overlapping pairs of atoms whose distances of separation were within a cutoff distance of 2 Å were searched for, and the glassy atoms were deleted. All structures were relaxed to zero pressure at 50 K for 200 ps prior to deformation and then loaded in uniaxial tension parallel to the z-direction using a strain rate of 4 × 10${}^{7}$ s${}^{-1}$. The stress normal to the loading direction was relaxed to allow for lateral contraction. Periodic boundary conditions were applied in all three directions. The atomic scale deformation mechanisms were analyzed by using the atomic von Mises strain [34], calculated by the OVITO analysis and visualization software [35]. The martensitic transformation process in the crystalline B2 inclusions and the dislocations activity in Cu phase were also monitored by the common neighbor analysis (CNA) scheme [36].

## 3. Results and Discussion

### 3.1. Effect of the Density of Inclusions

To investigate the effect the density of inclusions on the deformation behavior of Cu${}_{64}$Zr${}_{36}$ composites with B2 CuZr crystallites two structures have been prepared: one with 15 and another one with 9 rectangular inclusions of cross-sectional dimensions of 3.22 × 3.22 nm${}^{2}$ and an initial length of 19.4 nm. We will name these two composites 15n10uc and 9n10uc, respectively (here, uc is the abbreviation for CuZr B2 unit cells ≈ 0.322 nm). For better visualization of the competing deformation mechanisms in the B2 inclusions and the glassy matrix only those atoms of the glassy phase with a strain higher than 0.2 are displayed.

**Figure 1:**
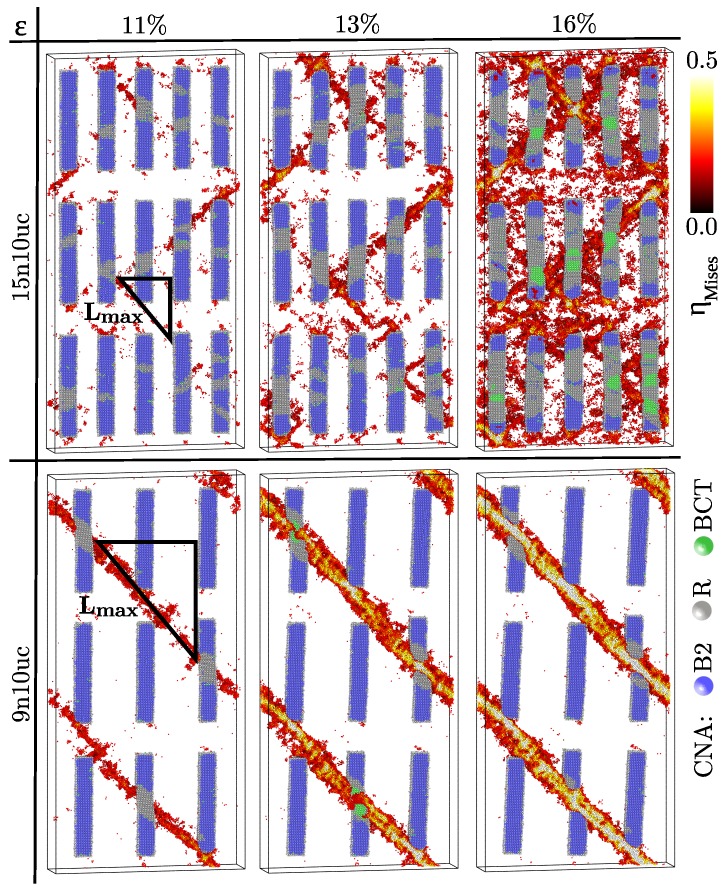
Evolution of the atomic von Mises strain and the results of the CNA in Cu${}_{64}$Zr${}_{36}$ metallic glass composites with 15 and 9 B2 CuZr precipitates of cross-sectional dimensions of 3.22 × 3.22 nm${}^{2}$ under tensile loading. All atomic positions are rescaled by affine transformation correcting for the macroscopic strain. In order to capture the competing deformation mechanisms in the B2 crystalline and glassy phases, only half of the structure and those atoms with an atomic strain higher than 0.2 are shown. The crystalline atoms are colored based on the local strain for values higher than 0.2. The drawn triangles are used to calculate the maximum free path for the shear band.

Upon tensile load, the MG composite with 15 inclusions (14.1 vol.%) shows homogeneous deformation. Initially, STZs mostly nucleate at the corners of the B2 inclusions, where the stress is concentrated, and around the soft amorphous-crystalline interface [25,37]. Once the B2 crystallites start to transform martensitically from the B2 structure to an intermediated R-phase (strain level of 11%) the strain/stress field is perturbed and STZs are activated where the material of the inclusions allow phase transformation (see Figure 1, upper panels). At a strain level of 13% the martensitic transformation in the B2 inclusions advances and more and more STZs are activated and percolate forming plastic zones (embryonic shear bands). Nevertheless, these plastic zones are confined between the crystalline inclusions and the formation of a critical shear front is hindered. Even at a strain level of 16% no critical shear band develops and the MG composite shows homogeneous plastic deformation. Moreover, along the directions where a higher density of STZs is activated and percolate the local stresses increase, causing the R-phase to martensitically transform to the body-centered-tetragonal (BCT) phase (green atoms in Figure 1). When two plastic zones intersect the local stress is drastically increased. This can mechanically dissolve (amorphize) the B2 inclusions locally (see Figure 1, upper panels).

When the number of B2 precipitates decreases to only 9 (8.4 vol.%) the response of the MG composite becomes extremely brittle and plastic deformation is highly localized in one dominant shear band (Figure 1, lower panels). The shear band formation is strongly related to the phase transformation in the crystalline inclusions: the martensitic transformation leads to shear initiation in the glassy matrix and the stress along the shear front induces martensitic transformation in the next shape memory crystals. However, in this case, the low number of B2 inclusions cannot block and confine the shear band. Two reasons can be responsible for the observed brittle-to-ductile transition: the low-volume fraction/size of the B2 inclusions or/and the critical shear band length [38]. For Cu${}_{64}$Zr${}_{36}$ metallic glass a threshold length of the shear band of about 20 nm was estimated [39]. Considering the system dimension along x-direction, the size of the inclusion and the fact that the shear band propagates at a 45 degree angle with respect to the loading direction one can calculate the maximum free path of the shear band following the simple geometrical construction shown in Figure 1. The longest free path that the shear band can follow in MG composites with 15 and nine inclusions is about 14 nm and 27 nm, respectively. Therefore, these values validate our results for the case of the MG composites with nine inclusions where a dominant shear band forms and propagates through the system. Nevertheless, for composites with 15 precipitates where the spacing between the inclusions is smaller than the critical shear band length, the plastic zones are confined by the inclusions and the deformation in the composites is mediated by both, STZ nucleation and percolation and martensitic phase transformation mechanisms.

### 3.2. Effect of the Size of the Inclusions

**Figure 2:**
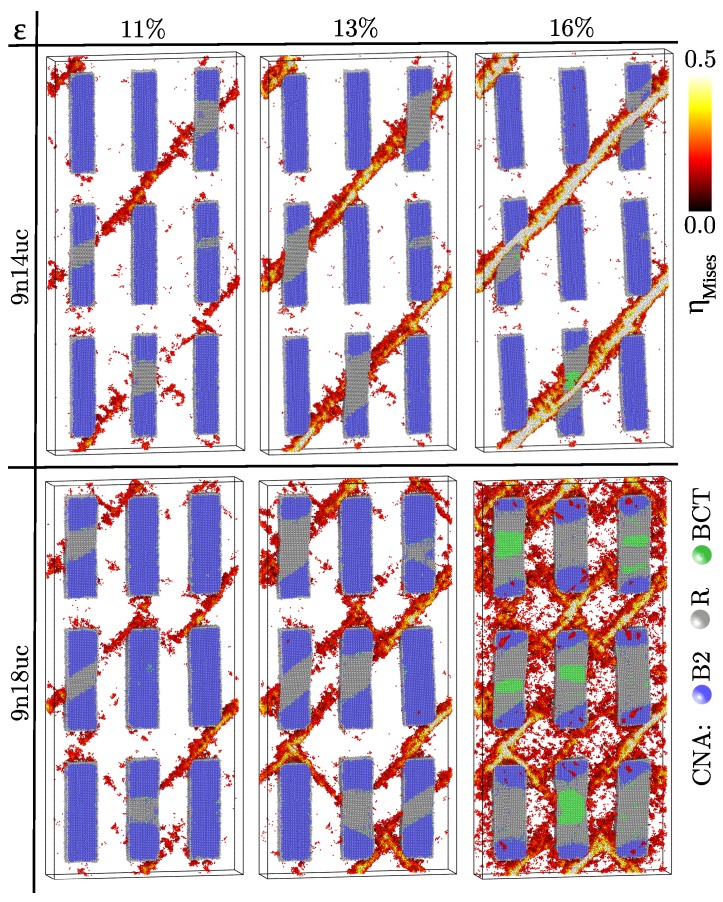
Evolution of the atomic von Mises strain and the results of the CNA in Cu${}_{64}$Zr${}_{36}$ metallic glass composites with 9 B2 CuZr precipitates of two cross-sectional dimensions of 4.5 × 4.5 and 5.8 × 5.8 nm${}^{2}$, respectively. All atomic positions are rescaled by affine transformation correcting for the macroscopic strain. In order to capture the competing deformation mechanisms in the B2 crystalline and glassy phases, only half of the structure and those atoms with an atomic strain higher than 0.2 are shown. The crystalline atoms are colored based on the local strain for values higher than 0.2.

To further differentiate between the effects of volume fraction and size of the B2 inclusions on the deformation behavior of MG composites with shape memory alloy we constructed another two composites with nine inclusions with cross-sectional dimensions of 4.5 × 4.5 and 5.8 × 5.8 nm${}^{2}$, respectively, while keeping the same length of 19.4 nm (named 9n14uc and 9n18uc composites, respectively). Figure 2 (upper panels) reveals that the plastic deformation in the 9n14uc composite is highly localized in a dominant shear band although this composite contains a high volume fraction of B2 phase (≈16.6 vol.%). The free-path for shearing is around 25 nm, providing the necessary condition for the formation of critical shear bands. Besides, there is also an effect of the size of the inclusions size. Under loading the shear band propagation is hindered for a while by the B2 inclusions while the morphology continuously changes. When comparing these findings to the case of composites with nine inclusions of 3.22 × 3.22 nm${}^{2}$ cross-section at a strain level of 13% the shear band in the 9n14uc composite is still confined by the B2 inclusions. Additionally, the martensitic deformation of the B2 crystals leads to a broadening of the shear band. However, at a strain level of 16% the shear band penetrates through the precipitates and a critical shear front occurs.

With further increasing the size of the B2 inclusions (27.6 vol.%) the 9n18uc composite shows homogeneous plastic deformation. Although the free path for shearing in this MG composite is long enough to form critical shear bands (≈23 nm) these planar defects are confined by the crystalline precipitates and the strain is homogeneously distributed in the whole composite on different slip planes carrying plastic deformation. Even at a strain level of 16% none of these shear bands becomes critical and there is a strong competition between the glassy and crystalline phases to accommodate the elastic energy. Hence, together with the formation of an organized pattern of multiple shear bands, the B2 inclusions are severely deformed and transform into the R and BCT phases. Both the distribution and the volume fraction of the B2 inclusions have a strong impact on the deformation mechanism of MG composites.

**Figure 3:**
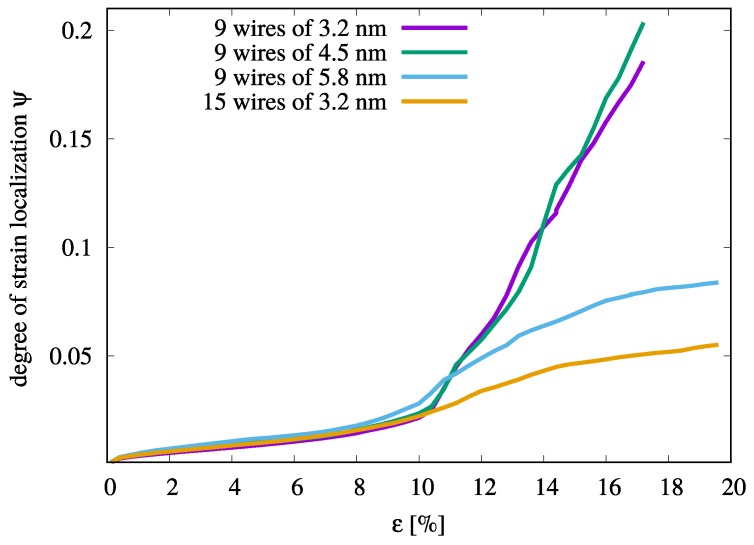
The degree of strain localization parameter, ψ, of the various Cu${}_{64}$Zr${}_{36}$ MG composites with B2 CuZr inclusions of different size and number during tensile deformation.

A quantitative interpretation of the aforementioned deformation mechanisms in MG composites can be derived by considering the strain localization parameter [40], $\psi =\sqrt{\frac{1}{N}\sum_{i=1}^{N}{\left(\eta_{i}^{Mises}-\eta_{ave}^{Mises}\right)}^{2}}$, where $\eta_{ave}^{Mises}$ is the average von Mises strain over all atoms in the simulation cell. A larger ψ value indicates larger variations in the atomic strain and a more localized deformation mode. As expected, the 15n10uc composite shows the lowest ψ values when compared to the composites with nine B2 inclusions (see Figure 3). Hence, this composite shows weak strain localization. Interestingly, although the 9n18uc composite also shows homogeneous plastic deformation the ψ value increases during the deformation process. The difference in the ψ value underlines the relationship between the ability of STZs to percolate and to form shear bands and the spacing between the B2 inclusions. In the 9n18uc composite, the sufficiently large free path for shearing ensures the formation of mature shear bands while in the 15n10uc composite only small plastic zones (embryonic shear bands) develop by STZ percolation. In contrast, the ψ parameter of the 9n10uc and 9n14uc composites increases exponentially upon continued loading once the strain level is above 11%. At this level of strain, one major shear band is formed that subsequently penetrates through the precipitates and mediates the plastic deformation across the samples for both composites.

### 3.3. Effects of Crystal Structure

**Figure 4:**
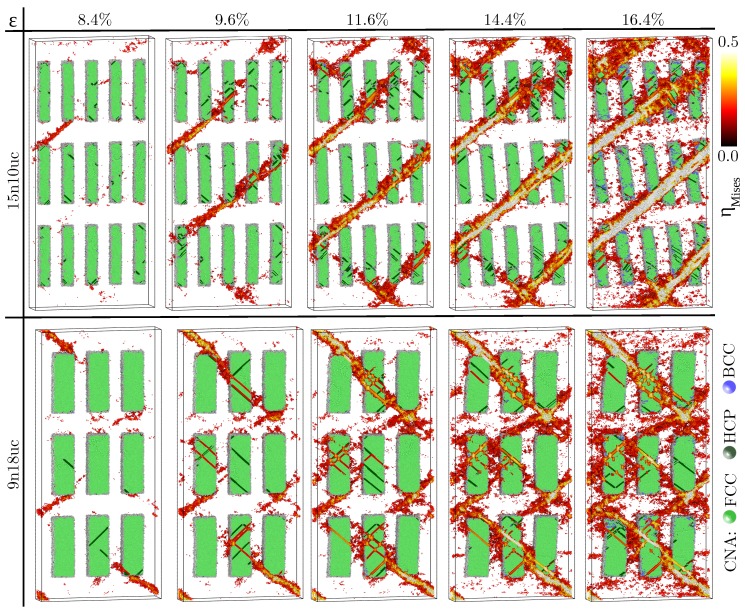
Evolution of the atomic von Mises strain and the results of the CNA in 15n10uc and 9n18uc composites with fcc Cu precipitates under tensile loading. All atomic positions are rescaled by affine transformation correcting for the macroscopic strain. In order to capture the competing deformation mechanisms in the fcc crystalline and glassy phases, only half of the structure and those atoms with an atomic strain higher than 0.2 are shown. The crystalline atoms are colored based on the local strain for values higher than 0.2.

To emphasize the superior mechanical properties of MG composites with shape memory crystals we compare their deformation mechanism to the one of composites with nanocrystalline inclusions which deform via dislocation activity. For this, we directed our attention to the two ductile MG composites, 15n10uc and 9n18uc, respectively, and replaced the B2 CuZr phase with pure face-centered cubic (fcc) Cu. Figure 4 shows the evolution of the atomic strain in the two MG composites together with the results of a common neighbor analysis in the fcc inclusions. First of all, we can see that MG composites simulated with the potential developed by Ward et al. [33] start yielding at strain level of 8%. Here, we should mention that the observed lower yield point for composites with fcc inclusions is related to the interatomic potential and should not be associated to the intrinsic properties of the crystalline inclusions.

Under loading, the composites with fcc inclusions show a more localized plastic deformation than the composites with shape memory phase. The fcc inclusions in the 15n10uc composite are too soft to block the shear band propagation cutting through the precipitates and slip transfer into the crystalline phase takes place. Initially, STZs are activated at the corners of the precipitates as well as by interactions with dislocations in the fcc inclusions [41,42,43] and percolate forming a dominant shear band. Moreover, the stress along the shear band overcomes the critical stress for heterogeneous nucleation of a dislocation in the precipitate and, hence, a higher density of dislocations along the shear front is visible. When the dislocations are perpendicular to the shear direction the shear band morphology is altered and the shear band broadens (see Figure 4). The plastic deformation in the 9n18uc composite becomes more homogeneous with increasing size of the fcc inclusions but still dominant shear bands form. The high volume fraction of crystalline phase is responsible for the formation of multiple shear bands. The lower barriers for STZ activation at amorphous-crystalline interfaces result in the activation of a high number of STZs and more homogeneous distribution of the plastic strain [27]. Altogether, the improved ductility originates mainly from the interaction between shear bands but only marginally from the interaction with crystalline precipitates. Even for large fcc inclusions the shear bands do propagate further and show slip transfer into the precipitates (see Figure 4).

**Figure 5:**
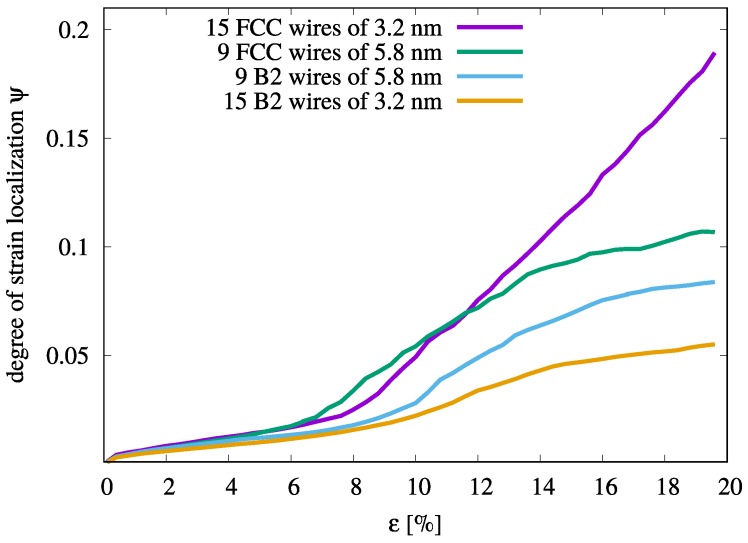
Variation of ψ parameter in 15n10uc and 9n18uc composites with CuZr B2 inclusions in comparison to the ψ parameter in two MG composites of the same configuration where the CuZr B2 phase was replace with fcc Cu inclusions.

The ψ parameter of the 15n10uc and 9n18uc composites with B2 CuZr or fcc Cu crystals is presented in Figure 5. It can clearly be seen that the ψ values of the composites with Cu inclusions increase drastically once yielding occurs. As already observed from the local atomic strain, the impact of the volume fraction of the soft crystalline phase on strain localization is noticeable and the ψ parameter of the 15n10uc composite (14.1 vol.%) shows the largest increase upon continued deformation. One should also remark the crossover of the curves for the 15n10uc and 9n18uc composites with fcc inclusions. A large volume fraction of crystalline precipitates coincides to a high surface ratio and consequently to a high volume fraction of amorphous-crystalline interfaces. Due to lowering activation barrier for STZ at the interfaces, the 9n18uc composite starts to yield first [25]. Nevertheless, these plastic zones are confined between the large fcc inclusions and under further loading, the ψ parameter of the 9n18uc composites shows a moderate increases (see Figure 5). Additionally, the crystalline phase is responsible for the difference in the ψ value encountered for 9n18uc composites. The lower values of ψ for the 9n18uc composite with B2 inclusions is due to the confinement effect of the shape memory phase.

## 4. Conclusions

In this paper, we have presented molecular dynamics simulations of the nanostructure–property relationships in shape memory metallic glass composites. Our results reveal that MG composites with a relatively low density of small B2 inclusions (14.1 vol.%) show homogeneous plastic deformation. The narrow spacing between the inclusions does not allow the STZs to percolate and form major shear bands. Hence, the plastic deformation in 15n10uc composite is mediated by the formation of embryonic shear bands and the martensitic phase transformation of B2 shape memory inclusions. Reducing the number (volume fraction) of B2 inclusions in the MG composites yields the ductile-to-brittle transition. The distance between inclusions in 9n10uc composites is within the critical shear band length which in turn leads to a highly localized deformation process. Otherwise, to ensure the arrest of shear bands and hence, homogeneous plastic deformation, a larger fraction of B2 inclusions beyond 28% is required. Substituting the shape memory inclusions with soft fcc Cu crystals renders more localized plastic deformation in the MG composites. Shear bands can cut through the Cu precipitates and a critical shear front occurs. Thus, by carefully choosing the density, size and spatial distribution of shape memory crystals, one can obtain MG composites that exhibit large tensile ductility for even a low-volume fraction of the shape memory phase.

## References

[1] He G., Eckert J., Löser W., Schultz L. (2003). Novel Ti-base nanostructure-dendrite composite with enhanced plasticity. Nat. Mater..

[2] Fan C., Inoue A. (1997). Improvement of mechanical properties by precipitation of nanoscale compound particles in Zr-Cu-Pd-Al amorphous alloys. Mater. Trans. JIM.

[3] Hays C.C., Kim C.P., Johnson W.L. (2000). Microstructure controlled shear band pattern formation and enhanced plasticity of bulk metallic glasses containing in situ formed ductile phase dendrite dispersions. Phys. Rev. Lett..

[4] Hays C.C., Kim C.P., Johnson W.L. (2001). Improved mechanical behavior of bulk metallic glasses containing in situ formed ductile phase dendrite dispersions. Mater. Sci. Eng. A.

[5] Eckert J., Das J., Pauly S., Duhamel C. (2007). Mechanical properties of bulk metallic glasses and composites. J. Mater. Res..

[6] Conner R., Dandliker R., Johnson W. (1998). Mechanical properties of tungsten and steel fiber reinforced Zr41.25Ti13.75Cu12.5Ni10Be22.5 metallic glass matrix composites. Acta Mater..

[7] Pauly S., Gorantla S., Wang G., Kuhn U., Eckert J. (2010). Transformation-mediated ductility in CuZr-based bulk metallic glasses. Nat. Mater..

[8] Chen M., Inoue A., Zhang W., Sakurai T. (2006). Extraordinary Plasticity of Ductile Bulk Metallic Glasses. Phys. Rev. Lett..

[9] Hofmann D.C., Suh J.Y., Wiest A., Duan G., Lind M.L., Demetriou M.D., Johnson W.L. (2008). Designing metallic glass matrix composites with high toughness and tensile ductility. Nature.

[10] Kim C.P., Oh Y.S., Lee S., Kim N.J. (2011). Realization of high tensile ductility in a bulk metallic glass composite by the utilization of deformation-induced martensitic transformation. Scr. Mater..

[11] Liu Z., Li R., Liu G., Song K., Pauly S., Zhang T., Eckert J. (2012). Pronounced ductility in CuZrAl ternary bulk metallic glass composites with optimized microstructure through melt adjustment. AIP Adv..

[12] Lee M., Lee C.M., Lee K.R., Ma E., Lee J.C. (2011). Networked interpenetrating connections of icosahedra Effects on shear transformations in metallic glass. Acta Mater..

[13] Wu F.F., Chan K., Li S.T., Wang G. (2014). Stabilized shear banding of ZrCu-based metallic glass composites under tensile loading. J. Mater. Sci..

[14] Barekar N., Pauly S., Kumar R., Kuhn U., Dhindaw B., Eckert J. (2010). Structure–property relations in bulk metallic Cu–Zr–Al alloys. Mater. Sci. Eng. A.

[15] Pauly S., Liu G., Gorantla S., Wang G., Kühn U., Kim D.H., Eckert J. (2010). Criteria for tensile plasticity in Cu-Zr-Al bulk metallic glasses. Acta Mater..

[16] Hofmann D.C. (2010). Shape Memory Bulk Metallic Glass Composites. Science.

[17] Wu Y., Xiao Y., Chen G., Liu C.T., Lu Z. (2010). Bulk Metallic Glass Composites with Transformation-Mediated Work-Hardening and Ductility. Adv. Mater..

[18] Zhang L., Zhang H., Li W., Gemming T., Wang P., Bönisch M., Şopu D., Eckert J., Pauly S. (2017). Beta-type Ti-based bulk metallic glass composites with tailored structural metastability. J. Alloys Compd..

[19] Hong S., Kim J., Park H., Kim Y., Suh J., Na Y., Lim K., Park J., Kim K. (2016). Gradual martensitic transformation of B2 phase on TiCu-based bulk metallic glass composite during deformation. Intermetallics.

[20] Zhang L., Narayan R., Fu H., Ramamurty U., Li W., Li Y., Zhang H. (2019). Tuning the microstructure and metastability of *β*-Ti for simultaneous enhancement of strength and ductility of Ti-based bulk metallic glass composites. Acta Mater..

[21] Hajlaoui K., Yavari A., LeMoulec A., Botta W., Vaughan F., Das J., Greer A., Kvick Å. (2007). Plasticity induced by nanoparticle dispersions in bulk metallic glasses. J. Non-Cryst. Solids.

[22] Luan Y.W., Li C.H., Han X.J., Li J.G. (2017). Plastic deformation behaviours of CuZr amorphous/crystalline nanolaminate: A molecular dynamics study. Mol. Simul..

[23] Seo J., Schryvers D. (1998). TEM investigation of the microstructure and defects of CuZr martensite. Part I: Morphology and twin systems. Acta Mater..

[24] Park H.J., Hong S.H., Park H.J., Kim Y.S., Kim J.T., Na Y.S., Lim K.R., Wang W.M., Kim K.B. (2018). Development of High Strength Ni–Cu–Zr–Ti–Si–Sn In-Situ Bulk Metallic Glass Composites Reinforced by Hard B2 Phase. Met. Mater. Int..

[25] Şopu D., Stoica M., Eckert J. (2015). Deformation behavior of metallic glass composites reinforced with shape memory nanowires studied via molecular dynamics simulations. Appl. Phys. Lett..

[26] Şopu D., Albe K., Eckert J. (2018). Metallic glass nanolaminates with shape memory alloys. Acta Mater..

[27] Albe K., Ritter Y., Şopu D. (2013). Enhancing the plasticity of metallic glasses: Shear band formation, nanocomposites and nanoglasses investigated by molecular dynamics simulations. Mech. Mater..

[28] Brink T., Peterlechner M., Rösner H., Albe K., Wilde G. (2016). Influence of Crystalline Nanoprecipitates on Shear-Band Propagation in Cu-Zr-Based Metallic Glasses. Phys. Rev. Appl..

[29] Plimpton S. (1995). Fast Parallel Algorithms For Short-Range Molecular-Dynamics. J. Comput. Phys..

[30] Mendelev M.I., Sordelet D.J., Kramer M.J. (2007). Using atomistic computer simulations to analyze x-ray diffraction data from metallic glasses. J. Appl. Phys..

[31] Sutrakar V.K., Mahapatra D.R. (2009). Stress-induced martensitic phase transformation in Cu-Zr nanowires. Mater. Lett..

[32] Sutrakar V.K., Mahapatra D.R. (2010). Single and multi-step phase transformation in CuZr nanowire under compressive/tensile loading. Intermetallics.

[33] Ward L., Agrawal A., Flores K. (2012). Rapid production of accurate embedded-atom method potentials for metal alloys. arXiv.

[34] Shimizu F., Ogata S., Li J. (2007). Theory of shear banding in metallic glasses and molecular dynamics calculations. Mater. Trans..

[35] Stukowski A. (2010). Visualization and analysis of atomistic simulation data with OVITO-the Open Visualization Tool. Model. Simul. Mater. Sci. Eng..

[36] Honeycutt J.D., Andersen H.C. (1987). Molecular dynamics study of melting and freezing of small Lennard-Jones clusters. J. Phys. Chem..

[37] Şopu D., Stukowski A., Stoica M., Scudino S. (2017). Atomic-Level Processes of Shear Band Nucleation in Metallic Glasses. Phys. Rev. Lett..

[38] Shi Y.F. (2010). Size-independent shear band formation in amorphous nanowires made from simulated casting. Appl. Phys. Lett..

[39] Şopu D., Soyarslan C., Sarac B., Bargmann S., Stoica M., Eckert J. (2016). Structure-property relationships in nanoporous metallic glasses. Acta Mater..

[40] Cheng Y.Q., Cao A.J., Ma E. (2009). Correlation between the elastic modulus and the intrinsic plastic behavior of metallic glasses: The roles of atomic configuration and alloy composition. Acta Mater..

[41] Wang Y.M., Li J., Hamza A.V., Barbee T.W. (2007). Ductile crystal line-amorphous nanolaminates. Proc. Natl. Acad. Sci. USA.

[42] Arman B., Brandl C., Luo S.N., Germann T.C., Misra A., Cagin T. (2011). Plasticity in Cu(111)/Cu46Zr54 glass nanolaminates under uniaxial compression. J. Appl. Phys..

[43] Brandl C., Germann T., Misra A. (2013). Structure and shear deformation of metallic crystalline–amorphous interfaces. Acta Mater..

